# Roles for ER:endosome membrane contact sites in ligand-stimulated intraluminal vesicle formation

**DOI:** 10.1042/BST20170432

**Published:** 2018-09-21

**Authors:** Louise H. Wong, Emily R. Eden, Clare E. Futter

**Affiliations:** Department of Cell Biology, UCL Institute of Ophthalmology, 11-43 Bath Street, London, EC1V 9EL, U.K.

**Keywords:** EGF receptor, intraluminal vesicle, membrane contact site, membrane trafficking, multivesicular body

## Abstract

Multivesicular endosomes/bodies (MVBs) sort membrane proteins between recycling and degradative pathways. Segregation of membrane proteins onto intraluminal vesicles (ILVs) of MVBs removes them from the recycling pathway and facilitates their degradation following fusion of MVBs with lysosomes. Sorting of many cargos onto ILVs depends on the ESCRT (Endosomal Sorting Complex Required for Transport) machinery, although ESCRT-independent mechanisms also exist. In mammalian cells, efficient sorting of ligand-stimulated epidermal growth factor receptors onto ILVs also depends on the tyrosine phosphatase, PTP1B, an ER-localised enzyme that interacts with endosomal targets at membrane contacts between MVBs and the ER. This review focuses on the potential roles played by ER:MVB membrane contact sites in regulating ESCRT-dependent ILV formation.

## Introduction

ESCRT (Endosomal Sorting Complex Required for Transport)-dependent sorting of ubiquitinated EGF (epidermal growth factor) receptor (EGFR) onto intraluminal vesicles (ILVs) of multivesicular endosomes/bodies (MVBs) plays an important role in down-regulation of EGFR signalling by removing the catalytic domain of the receptor from the cytoplasm and targeting the receptor for degradation. EGF stimulation of EGFR promotes not only the incorporation of the receptor onto ILVs but also the formation of the ILVs themselves [1]. EGF-stimulated ILV formation depends on the ESCRT machinery since depletion of ESCRT components inhibits EGF-stimulated ILV formation [2]. Furthermore, a poorly ubiquitinated EGFR that is impaired in its ability to recruit ESCRT0 is unable to promote EGF-stimulated ILV formation [3]. Depletion of ubiquitinated cargo in yeast has been shown to inhibit ILV formation [4], and so, the presence of increased ubiquitinated cargo (EGFR) on the limiting membrane of MVBs following EGF stimulation could contribute to the increased ILV formation. However, this is unlikely to be the sole explanation of enhanced ILV formation in EGF-stimulated cells because we have identified two proteins, annexin A1 and protein tyrosine phosphatase 1B (PTP1B), that are not normally considered part of the ESCRT machinery, but are absolute requirements for EGF-stimulated ILV formation [1,5].

## Roles of annexin A1 and PTP1B in EGF-stimulated ILV formation

In studies that preceded the identification of the ESCRT machinery, the activity of the EGFR kinase was shown to be necessary for efficient targeting of EGFR onto ILVs [6], leading us to search for substrates of the EGFR kinase associated with MVBs. We showed that the calcium and phospholipid-binding protein, annexin A1, associated with EGFR-containing MVBs where it underwent EGF-stimulated phosphorylation [7] and found that annexin A1 depletion or knockout had no effect on ILV formation in resting cells, but abolished EGF-stimulated ILV formation [1]. EGF-stimulated ILV formation could be rescued by re-expression of wild-type annexin A1 but not a mutant lacking the single tyrosine phosphorylation site in the N-terminus of the protein. Annexin A1 has been shown to mediate membrane aggregation *in vitro* [8], leading us to suggest models whereby annexin A1 molecules on opposing membranes of deeply invaginating buds might help to promote vesicle scission [9]. However, a subsequent investigation into the role of the protein tyrosine phosphatase, PTP1B, which dephosphorylates EGFR, led us to a quite different view of the role of annexin A1.

**Figure 1.**
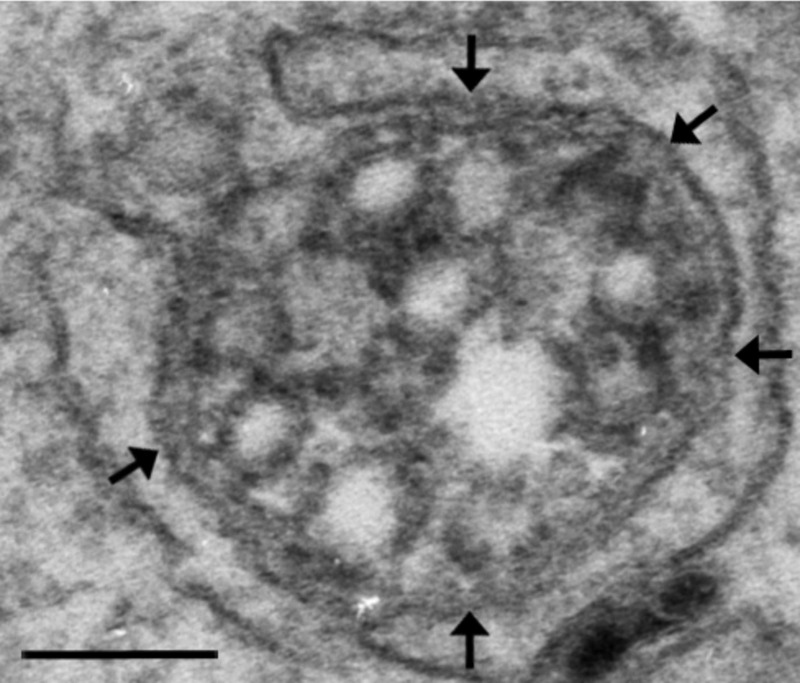
Expression of a substrate-trapping mutant of PTP1B promotes the formation of extended membrane contacts between MVBs and the ER. HeLa cells transfected with substrate-trapping mutant PTP1B were stimulated with EGF conjugated to horseradish peroxidase which generates the formation of electron dense reaction product within EGFR-containing MVBs. An extended contact (arrows) between the ER and the MVB-limiting membrane is shown. Bar: 100 nm.

PTP1B is embedded in the cytoplasmic face of the ER and yet Haj et al. [10] showed that endocytosed EGFR interacts with PTP1B, raising the question of how these two proteins on different organelles interact. By expressing a substrate-trapping mutant of PTP1B that prolongs what would normally be a very transient interaction between the phosphatase and its substrate, we were able to show that this interaction occurs at membrane contact sites between the ER and EGFR-containing MVBs [5] (Figure 1). The ER forms membrane contacts with virtually every organelle within the cell and the last decade has seen an explosion in our understanding of membrane contact site biology and in the formation and function of contacts between the ER and the endocytic pathway (Table 1). Depleting cells of PTP1B not only prolonged phosphorylation of endocytosed EGFR but also, like annexin A1 depletion, inhibited EGF-stimulated ILV formation [5]. Given the potential for annexin A1 to aggregate membranes, this raised the possibility that annexin A1 may regulate membrane contact site formation between the ER and EGFR-containing MVBs and the further intriguing possibility that membrane contact sites are required for EGF-stimulated ILV formation.

**Table 1:** Established roles of ER contacts with endocytic organelles

Established roles of ER contacts with endocytic organelles*		Proteins/complexes involved in contact formation and/or function
Regulation of receptor tyrosine kinases	ER-localised phosphatase, PTP1B, dephosphorylates endocytosed EGFR at annexin A1-dependent contacts.	PTP1B:EGFR [5] annexin A1:S100A11 [11]
Cholesterol transfer ER to endosome	In the absence of LDL, ORP1L regulates the transfer of cholesterol from the ER to the endosome at annexin A1-dependent contact sites.	ORP1L:VAP annexin A1:S100A11 [11]
	StARD3 is able to mediate both ER:endosome contacts and transfer cholesterol from the ER to the endosome.	StARD3:VAP [44]
Endosomal trafficking (neurite outgrowth)	ER-localised protrudin interacts with Rab7 to regulate loading of kinesin-1-promoting plus-end transport towards the cell periphery.	Protrudin:Rab7 [13]
Endosomal positioning	Low endosomal cholesterol promotes ORP1L:VAP binding to induce ER:endosome contact formation where VAP interacts with Rab7:RILP. Consequently, dynactin is removed, promoting microtubule plus-end directed movement.	VAP:ORP1L:Rab7:RILP:dynein(p150^Glued^):HOPS [14]
	ER-localised E3 ubiquitin ligase RNF26 recruits the ubiquitin scaffold, p62/SQSTM1, to interact with ubiquitin-binding domains of vesicle adaptors, restricting vesicles to the perinuclear region.	RNF26:SQSTM1:vesicle adaptors [50]
Endosome fission	VAP interacts with retromer SNX2 subunit and OSBP to regulate PI4P dynamics affecting actin nucleation and retromer function.	VAP:SNX2 VAP:OSBP [51]
	ER:endosome contacts occur at FAM21-positive sorting domains immediately before fission events that mediate budding of tubules for recycling.	FAM21 [52]

* Examples where the membrane contact has been unequivocally shown to be necessary.

## Annexin A1 regulates membrane contact site formation between the ER and EGFR-containing MVBs

We have previously shown that EGFRs are trafficked in a subset of MVBs [1], but membrane contacts with the ER are not confined to this subset of MVBs. We and others have found that the ER makes contacts with multiple components of the endocytic pathway [5,11–15], with the frequency/extent of contacts increasing with progression along the pathway to the lysosome [16]. We have further shown that ER contacts with EGFR-containing MVBs, non-EGFR-containing MVBs and lysosomes are biochemically distinct, since depletion of annexin A1, or its ligand S100A11, has no effect on contacts with non-EGFR–MVBs and lysosomes, but dramatically impairs the formation of ER contacts with EGFR-containing MVBs [11]. Annexin A1 forms a tetrameric complex with S100A11 and both components localise to the contacts, consistent with this complex tethering the contacts. Also consistent with a role for annexin A1 as a tether is the increased contact site formation upon overexpression of wild-type annexin A1 but not a mutant lacking the EGF-stimulated tyrosine phosphorylation site [11]. Conversely, overexpression of a Y21E mutant of annexin A1 that may be a ‘phospho-mimetic’ mutation induced the formation of particularly extended contacts [11]. Mutational analysis of annexin A1 reveals similar requirements for membrane contact site and EGF-stimulated ILV formation, consistent with EGF-stimulated ILV formation being mediated through phosphorylation of annexin A1 and resulting promotion of membrane contact site formation between the ER and EGFR-containing MVBs [11].

## How do membrane contact sites regulate ILV formation?

### PTP1B-mediated dephosphorylation of endosomal targets

ESCRT-mediated sorting of EGFR begins with the concentration of ubiquitinated EGFR in clathrin-coated domains on the limiting membrane of MVBs. Clathrin coats and membrane contact sites with the ER are found on the same MVB, sometimes immediately adjacent to each other [17]. The presence of the clathrin coat may be a physical barrier to contact site formation, but the temporal relationship between the clathrin coat and the membrane contact remains unclear. Both recruitment of ubiquitinated EGFR and clathrin into clathrin-coated domains depends on the ESCRT0 component, Hrs [18,19]. Hrs, and another ESCRT0 component, STAM1/2, are phosphorylated in response to growth factor stimulation [20] and intriguingly, both Hrs and STAM1/2 are also subject to dephosphorylation by PTP1B [5,21]. Although Hrs and STAM1/2 are soluble proteins and so would not necessarily require a contact site to interact with PTP1B, Hrs phosphorylation requires its recruitment to endosomes [22,23] and so would be well placed to be dephosphorylated by PTP1B at ER:endosome contact sites. The relationship between Hrs phosphorylation state and its ability to recruit clathrin would be an important indicator of the relationship between contact sites and the clathrin coat. EGF-stimulated phosphorylation of Hrs was not required for clathrin binding *in vitro* [24], but whether Hrs phosphorylation state can directly modulate clathrin binding/dissociation is unclear.

The role of phosphorylation/dephosphorylation of Hrs and STAM1/2 in regulating activity of the ESCRT0 complex is also not clear. Several studies indicate that the major EGF-stimulated tyrosine phosphorylation sites within Hrs (Y329 and Y334) are important for efficient progression of EGFR along the endocytic pathway to the lysosome [23,25] and STAM2 phosphorylation mutants displayed altered endosomal localisation [21]. The ubiquitin-interacting motif of Hrs, which binds ubiquitinated cargo, is required for EGF-stimulated Hrs phosphorylation [23] and can itself become ubiquitinated [26], preventing its interaction with ubiquitinated cargo. The relationship between Hrs ubiquitination and phosphorylation remains to be fully established, but when Hrs ubiquitination was enhanced by overexpression of the ubiquitin ligase, c-Cbl, Hrs became hyperphosphorylated [25]. Hrs phosphorylation promotes its release from the endosomal membrane [22,27] and could represent a means to release ubiquitinated Hrs from the MVB-limiting membrane to allow replacement by non-ubiquitinated Hrs and further rounds of ubiquitinated cargo sorting. Dephosphorylation of Hrs at membrane contact sites could represent an alternative pathway whereby Hrs becomes dephosphorylated and de-ubiquitinated *in situ* at the contact without a requirement for membrane dissociation and subsequent proteasomal degradation. This hypothesis would predict the presence at the contacts of deubiquitinating enzymes, such as USP8. Loss/depletion of USP8 leads to proteasomal degradation of STAM and Hrs [28–30], but whether this enzyme operates at membrane contact sites is unclear. PTP1B-mediated Hrs and/or STAM dephosphorylation could also be required for progression of cargo through the later ESCRT pathway to ILV formation either by affecting the ability of Hrs to recruit the ESCRT1 component, Tsg101, or the ability of STAM to bind another phosphatase, HD-PTP. HD-PTP co-ordinates release of ESCRT0 with recruitment of the deubiquitinating enzyme UBPY/USP8 and ESCRTIII through competitive interactions [31] and so is an important link between ESCRT0 and ILV formation.

### Provision of ER-derived cholesterol to support ILV formation when low-density lipoprotein-derived cholesterol is low

**Figure 2.**
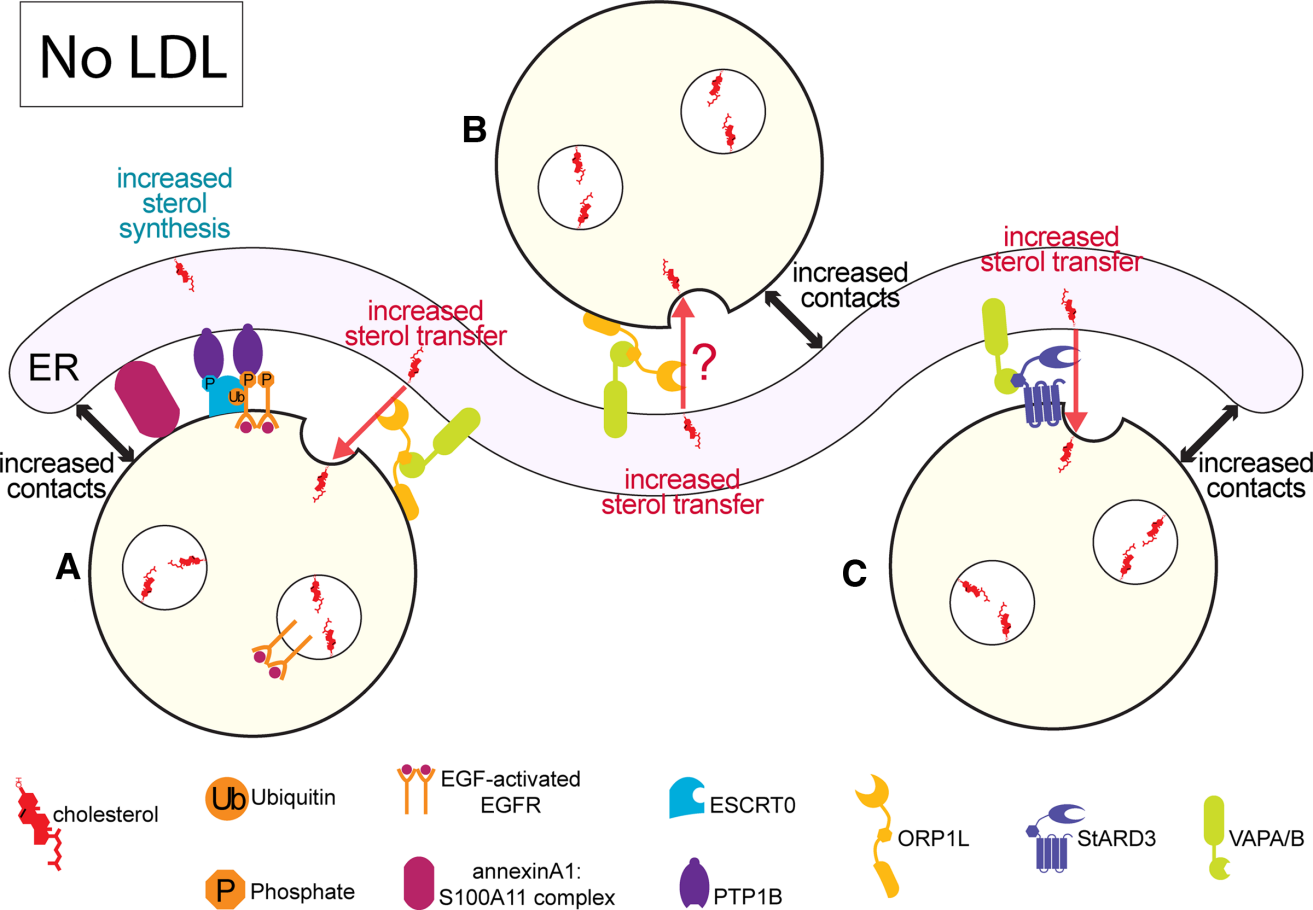
Illustration of ILV formation in the absence of LDL-derived cholesterol. Without access to LDL, cells increase cholesterol synthesis in the ER to meet cellular demand for cholesterol. ILVs contain high concentrations of cholesterol and the formation of ILVs in the absence of endocytosed LDL requires ER-derived cholesterol. In these conditions, ER:endosome membrane contacts expand to provide sites for cholesterol transfer. (**A**) In EGFR-positive MVBs, ER:endosome membrane contacts are formed by annexin A1:S100A11 heterotetramers. These contacts allow PTP1B to interact with EGFR and ESCRT0. ORP1L is also recruited to the annexin A1-dependent contacts to facilitate cholesterol transfer from the ER to endosomes via interaction with VAP on the ER. (**B**) ORP1L is also recruited to EGFR-negative endosomes where it expands contacts with the ER via interaction with VAPs and is potentially well placed to carry out cholesterol transfer activity. (**C**) StARD3 is present on endosomes distinct from those positive for ORP1L. StARD3 has been shown to promote expansion of ER:endosome contacts via interaction with VAPs and to transfer cholesterol from the ER to the endosomes to support ILV formation. Abbreviations: EGF, epidermal growth factor; EGFR, EGF receptor; ESCRT, Endosomal Sorting Complex Required for Transport; ILV, intraluminal vesicle; LDL, low-density lipoprotein; ORP1L, OSBP-related protein 1-like; P, phosphate group; PTP1B, protein tyrosine phosphatase 1B; StARD3, Steroidogenic Acute Regulatory lipid transfer protein 3; Ub, ubiquitin; VAPA/B, VAMP-associated protein A/B.

Cholesterol is a cell-wide lipid but its distribution is uneven across different organelle membranes. The plasma membrane is particularly cholesterol enriched with ∼40% mol compared with the site of synthesis, the ER, where it is found at 5% mol [32]. Even within a single MVB, cholesterol is found at contrasting levels; ILVs harbour 85% of total MVB cholesterol [33]. Cholesterol within the endocytic pathway is usually supplied by low-density lipoprotein (LDL), an extracellular source of cholesterol internalised by receptor-mediated endocytosis. In the absence of LDL, sterol synthesis in the ER is up-regulated through cleavage of sterol regulator element-binding protein allowing its transport to the nucleus to activate transcription of genes involved in sterol synthesis and uptake [34,35]. The high cholesterol content of ILVs suggests a role for cholesterol in ILV formation. Indeed, at least 10% mol cholesterol is necessary for *in vitro* ESCRTII self-assembly and associated lipid-phase separation that likely facilitates budding and scission for ILV formation [36]. Consistent with a cholesterol dependency for ILV formation, we demonstrated a substantial decrease in EGF-stimulated ILV formation upon cholesterol depletion [11]. However, it was necessary to deplete cells of both LDL and ER-derived cholesterol, implying that in the absence of LDL ER-derived cholesterol supports EGF-stimulated ILV formation [11]. The potential role of ER:endosome contacts in lipid exchange raised the possibility that, when endosomal cholesterol is low, ILV formation could be supported by transfer of ER-derived cholesterol at the annexin A1-dependent ER:endosome contacts described above. Consistently, in the absence of LDL, the cholesterol content of EGFR-containing MVBs is dependent on the extent of these contacts. Extending the contacts by expressing ‘phospho-mimetic’ annexin A1 caused greater MVB cholesterol accumulation, whereas depleting annexin A1 reduced MVB cholesterol content [11]. Thus, when there is a necessity for ER-derived cholesterol, annexin A1-tethered contact sites enable the flow of cholesterol from the ER to the endosome to support ILV formation (Figure 2).

Lipid transfer between two membranes can occur via lipid transfer proteins which can protect a lipid from an aqueous cytosol in transit from donor to acceptor membrane [37]. ORP1L is a member of the family of OSBP (oxysterol-binding protein)-related proteins (ORPs) that have a conserved OSBP-related domain that can bind lipids. The founding member of the ORP family, OSBP, was originally identified by its ability to bind oxysterols, but further studies have found that many ORPs are able to bind cholesterol [38] including ORP1L [39]. Overexpression of ORP1L expands ER:endosome contacts via a sterol-sensitive interaction with ER-localised VAPs (VAMP-associated protein) [14] via a conserved FFAT motif found in many VAP-binding proteins [40]. Although Eden et al. [11] found that depletion of ORP1L or VAPs had little effect on the formation of contacts between EGFR-containing MVBs and the ER, ORP1L depletion did reduce both the amount of cholesterol and the number of ILVs present in the MVBs in the absence of LDL [11]. Cholesterol content and ILV formation could be restored by re-expressing wild-type but not mutant ORP1L deficient in VAP binding. ORP1L is recruited to endosomes via Rab7 [41], a marker of mature MVBs and lysosomes, and yet ILV formation in EGFR-containing MVBs begins early in the endocytic pathway, rapidly after EGFR stimulation. Intriguingly, a pool of ORP1L was found to associate with EGFR-containing MVBs, regardless of cholesterol status, where it was recruited to membrane contact sites with the ER in the absence of LDL [11]. Thus, the ORP1L:VAP interaction that was previously shown to be promoted by low sterol conditions [14] appears necessary for cholesterol transport from the ER to EGFR-containing MVBs at annexin A1-dependent contacts to support ILV formation (Figure 2).

Whilst ORP1L is involved in ER cholesterol transfer to EGFR-containing endosomes, another lipid transfer protein, StARD3, has been implicated in transfer of cholesterol from the ER to endosomes in non-EGF-stimulated cells (Figure 2). StARD3 is a member of a major family of lipid transfer proteins, many of which have been shown to transfer sterol via its conserved StART (Steroidogenic Acute Regulatory lipid Transfer protein) domain, a helix-grip fold with a lipid-binding cavity [42]. ORP1L and StARD3 have been shown to be recruited to separate populations of endosomes [43] but, like ORP1L, StARD3 has a FFAT motif, able to interact with VAPs on the ER and its overexpression promotes ER contact site formation with MVBs [15]. In a recent study, StARD3 overexpression was shown to increase endosomal cholesterol at the expense of the plasma membrane and increase numbers of internal membranes including ILVs, within MVBs of non-EGF-stimulated cells [44]. These effects depended on the StART domain of STARD3 and the ability of StARD3 to bind VAP, strongly suggesting that StARD3 transfers ER-derived cholesterol at ER:endosome contact sites. In this study, *in vitro* assays were established that directly tested the activity of StARD3 in cholesterol transfer. Although soluble StARD3 (without the transmembrane domain) was able to transfer cholesterol between donor and acceptor liposomes, the speed of the activity was 20 times faster when StARD3 was anchored to ‘endosome-like’ acceptor liposomes and VAP was anchored to ‘ER-like’ donor liposomes. Under these conditions the liposomes aggregated, modelling contact site formation. Mutation of the FFAT motif which regulates VAP interaction, abolished liposome aggregation and reduced sterol transfer to speeds comparable to soluble StARD3, consistent with fast cholesterol transfer occurring at donor:acceptor liposome contacts.

Recently, MOSPD2 (motile sperm domain-containing protein 2) was identified as another ER-resident protein that, like VAP, binds FFAT motif-containing proteins (including ORP1L and STARD3) and is required for ER–endosome contact site formation. The function of MOSPD2-regulated contacts is not yet understood, but MOSPD2 contains a CRAL-TRIO domain, suggesting a likely role in lipid transport [45].

## Potential future directions

Although the role of ESCRT0 in sorting ubiquitinated cargo for incorporation onto ILVs is relatively well established, the way in which this activity is regulated by phosphorylation/dephosphorylation is far from clear. ESCRT0 phosphorylation occurs on endosomes but whether or not this happens within clathrin-coated domains is unknown. ESCRT0 dephosphorylation likely occurs at ER:endosome contact sites, but the relationship of the contacts with clathrin-coated domains is unclear. Both the clathrin coat and ER:endosome membrane contact sites must be disassembled before scission of the ILV but how this is regulated is largely unknown, although the AAA ATPase, Vps 4, has been implicated in clathrin coat dissociation [46]. At least one component of ESCRT0, Hrs, has other less well-characterised functions that appear to be independent of the later ESCRT machinery. Hrs has been implicated in recycling of heterotrimeric G protein-coupled receptors [47], a function that is apparently opposed to ESCRT-dependent sorting to the lysosome. Hrs (and Vps4) have also been implicated in egress of LDL-derived cholesterol from endosomes to the ER [48,49], i.e. in the opposite direction to the transport of ER-derived cholesterol to endosomes at membrane contact sites to support ILV formation (described above). Since ER–endosome contact sites (and, most likely, PTP1B-mediated dephosphorylation of Hrs) are increased under conditions of low LDL [11,14], there may be reciprocal regulation of Hrs function and the sterol environment of the endocytic pathway. It is tempting to speculate that regulation of Hrs phosphorylation at membrane contact sites between the endocytic pathway and the ER co-ordinates the multiple ESCRT-dependent and -independent roles of Hrs. Hopefully, tools that allow not only contact site assembly but also disassembly to be manipulated will allow their role in regulation of ESCRT0 phosphorylation and function to be established.

## Abbreviations

EGF: epidermal growth factor
EGFR: EGF receptor
ESCRT: Endosomal Sorting Complex Required for Transport
ILVs: intraluminal vesicles
LDL: low-density lipoprotein
MOSPD2: motile sperm domain-containing protein 2
MVBs: Multivesicular endosomes/bodies
OSBP: oxysterol-binding protein
ORP: OSBP-related proteins
PTP1B: protein tyrosine phosphatase 1B
StART: Steroidogenic Acute Regulatory lipid Transfer protein
VAP: VAMP-associated protein
